# Supplementary material for: In vivo RNA interactome profiling reveals 3’UTR-processed small RNA targeting a central regulatory hub
Source: Nat Commun. 2023 Dec 7;14:8106. doi: 10.1038/s41467-023-43632-1 (PMC10703908; doi:10.1038/s41467-023-43632-1)
Supplement: Supplementary file 1 — Supplementary Information [file 41467_2023_43632_MOESM1_ESM.pdf]

In vivo RNA interactome profiling reveals 3’UTR-processed small RNA targeting a central regulatory hub

Fang Liu, Ziying Chen, Shuo Zhang, Kejing Wu, Cheng Bei, Chuan Wang, Yanjie Chao

Supplementary Information

- Supplementary Figure 1-8
- Supplementary Data 1-7

Supplementary Figure 1

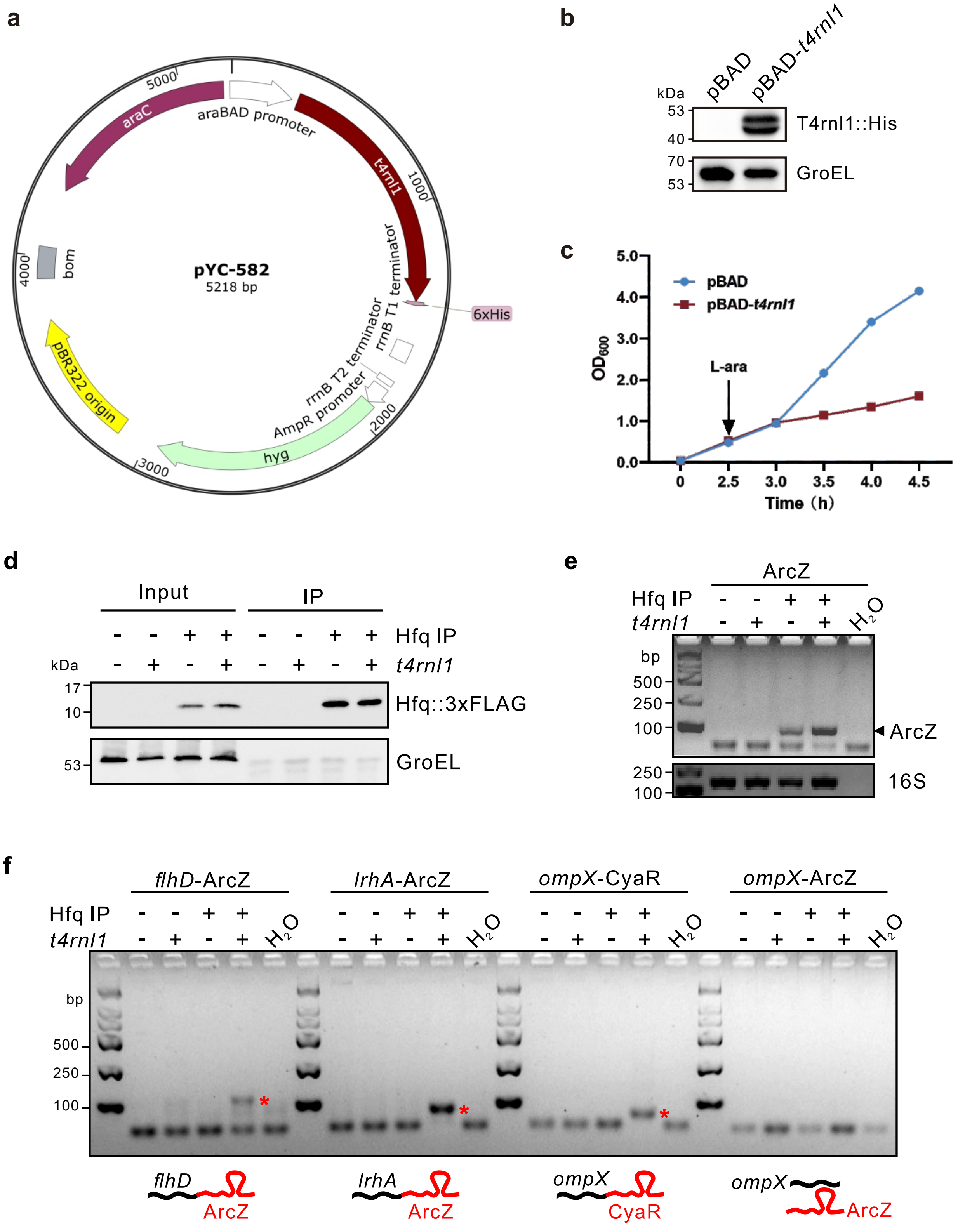

Supplementary Fig. 1 sRNA-target interactions detected by iRIL-seq. a Plasmid map for pBAD-*t4rnl* (pYC582). **b** Western blot detection of T4 RNA ligase 1 expressed *in vivo*. *Salmonella* strains carrying an empty vector or plasmid pYC582 were grown in LB to an OD_{600} of 2.0. Bacterial cultures were supplemented with 0.2% L-arabinose for 30 min. Total proteins were analyzed by Western blotting using an anti-HIS antibody. GroEL serves as a loading control. **c** Potential toxic effect of T4 RNA ligase during the log phase of *Salmonella* growth in LB. The T4 RNA ligase was induced when bacteria were grown to an OD_{600} of 0.5. Bacterial growth (OD_{600}) was monitored every 30 minutes. **d** Western blot verification of Hfq-coIP pulldown. *Salmonella* WT and Hfq::FLAG strains carrying an empty vector or plasmid pYC582 were grown in LB to an OD_{600} of 1.5. Bacteria were treated with 0.2% L-arabinose for 30 min and grown to OD 2.0. Cells were collected and subjected to iRIL-seq experiments. Input, total proteins from bacterial lysates. IP, proteins after immunoprecipitation with anti-FLAG antibody. Blot shown is representative of n = 2 biological replicates. **e** Verification of the ArcZ sRNA in iRIL-seq samples using reverse transcription and PCR (RT-PCR). The RNA samples collected from panel **c** were reverse transcribed using random hexamer; the resulting cDNA samples were amplified using ArcZ specific oligos. H_2O served as a no template control. 16S rRNA served as internal control. **f** Detection of known sRNA-target chimeras (red asterisks). cDNA samples from panel **d** were used for PCR validation. Chimeras were detected using forward primers sense to the target mRNAs, and reverse primers antisense to sRNAs. H_2O served as a no template control. Source data are provided as a Source Data file.

Supplementary Figure 2

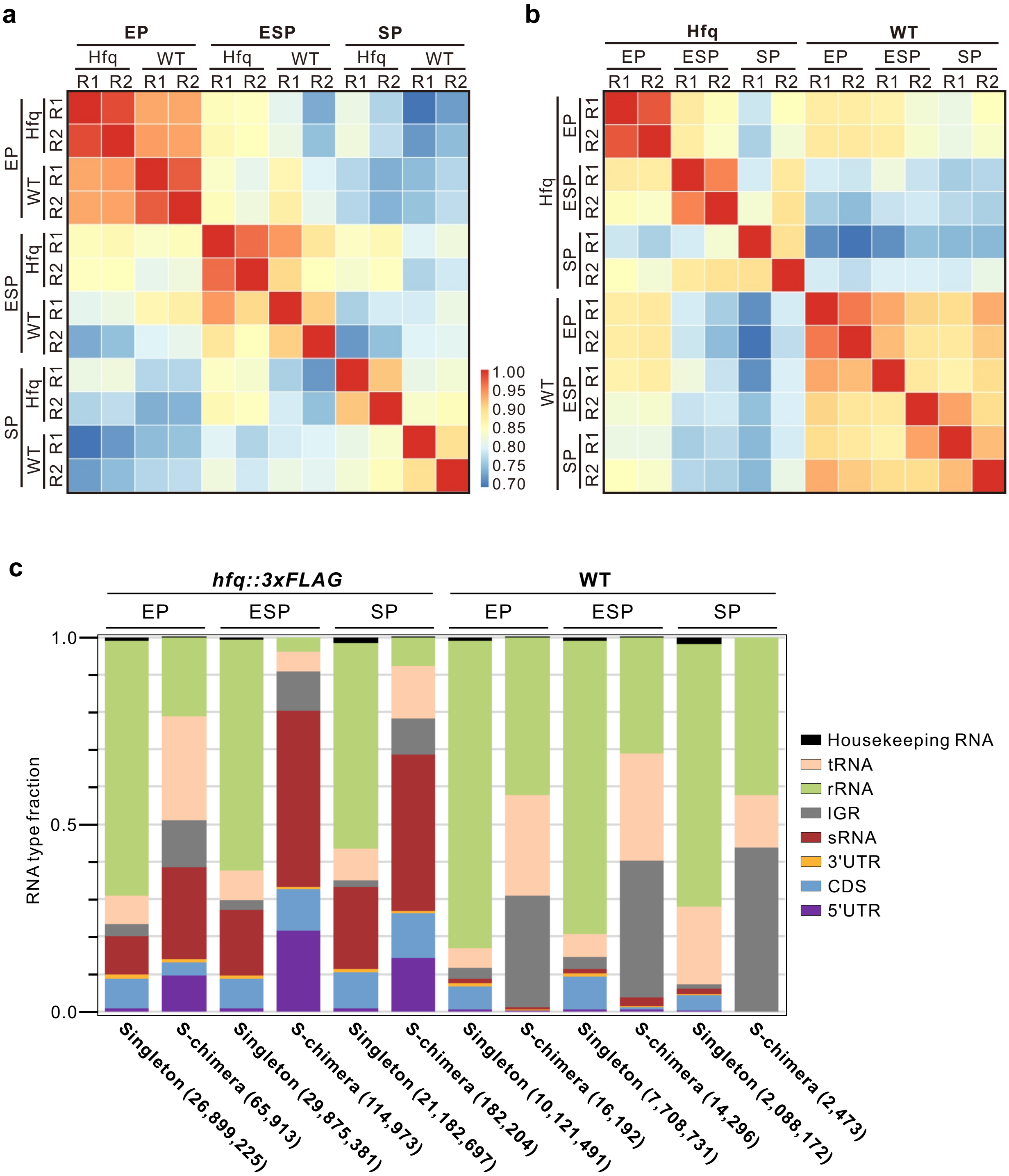

Supplementary Fig. 2 Data analysis of iRIL-seq datasets at three growth stages. a cDNA libraries containing singleton fragments were clustered using correlation coefficients. **b** cDNA libraries containing S-chimera fragments were clustered using correlation coefficients. **c** Distribution of each transcript type for singleton and S-chimeric fragments at three growth stages. The total number of sequenced fragments is denoted in parentheses. Housekeeping RNAs: RnpB, SsrS, Ffs, SsrA. IGR: intergenic region.

Supplementary Figure 3

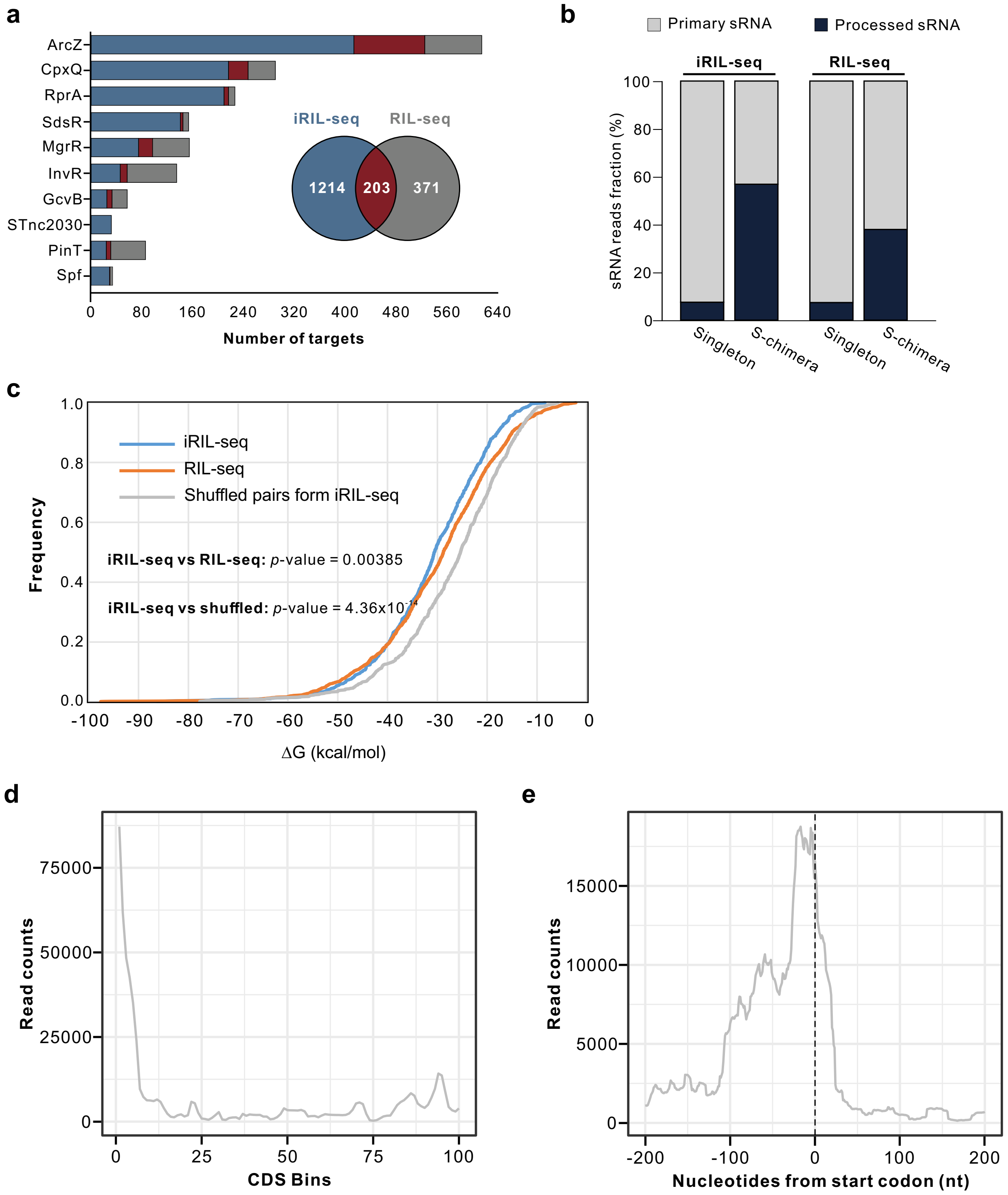

Supplementary Fig. 3 Comprehensive comparison between iRIL-seq and RIL-seq. a Comparison of the sRNA-mRNA interactions found by iRIL-seq (three growth stages) and RIL-seq data (ESP at OD 2.0). The bars indicate the numbers of predicted targets for ten sRNAs that have most predicted targets. Venn diagram (inset) shows the overlap of all predicted targets for these ten sRNAs between the two datasets. **b** Processed sRNAs (Supplementary Data 6) were enriched in S-chimeras in both iRIL-seq and RIL-seq. **c** Comparison of RNA–RNA interaction strength (ΔG, kcal/mol) for all RNA–RNA interactions in S-chimeras. Energies of RNA-RNA interactions were calculated by RNADuplex. Pairs of interacting RNAs in iRIL-seq were randomly shuffled. A two-sided Kolmogorov–Smirnov test was used to calculate *p*-values. **d** S-chimeric reads per million total reads detected by iRIL-seq and RIL-seq. **e** S-chimeras per million total reads detected by iRIL-seq and RIL-seq. **f** Distribution of RNA1 fragments in S-chimeras over all protein-coding genes in iRIL-seq. Each gene was divided in 100 bins. The number of RNA1 fragments mapped to each bin was calculated. **g** Distribution of RNA1 fragments in S-chimeras at start codons in iRIL-seq. Dashed line indicates the position of start codons.

Supplementary Figure 4

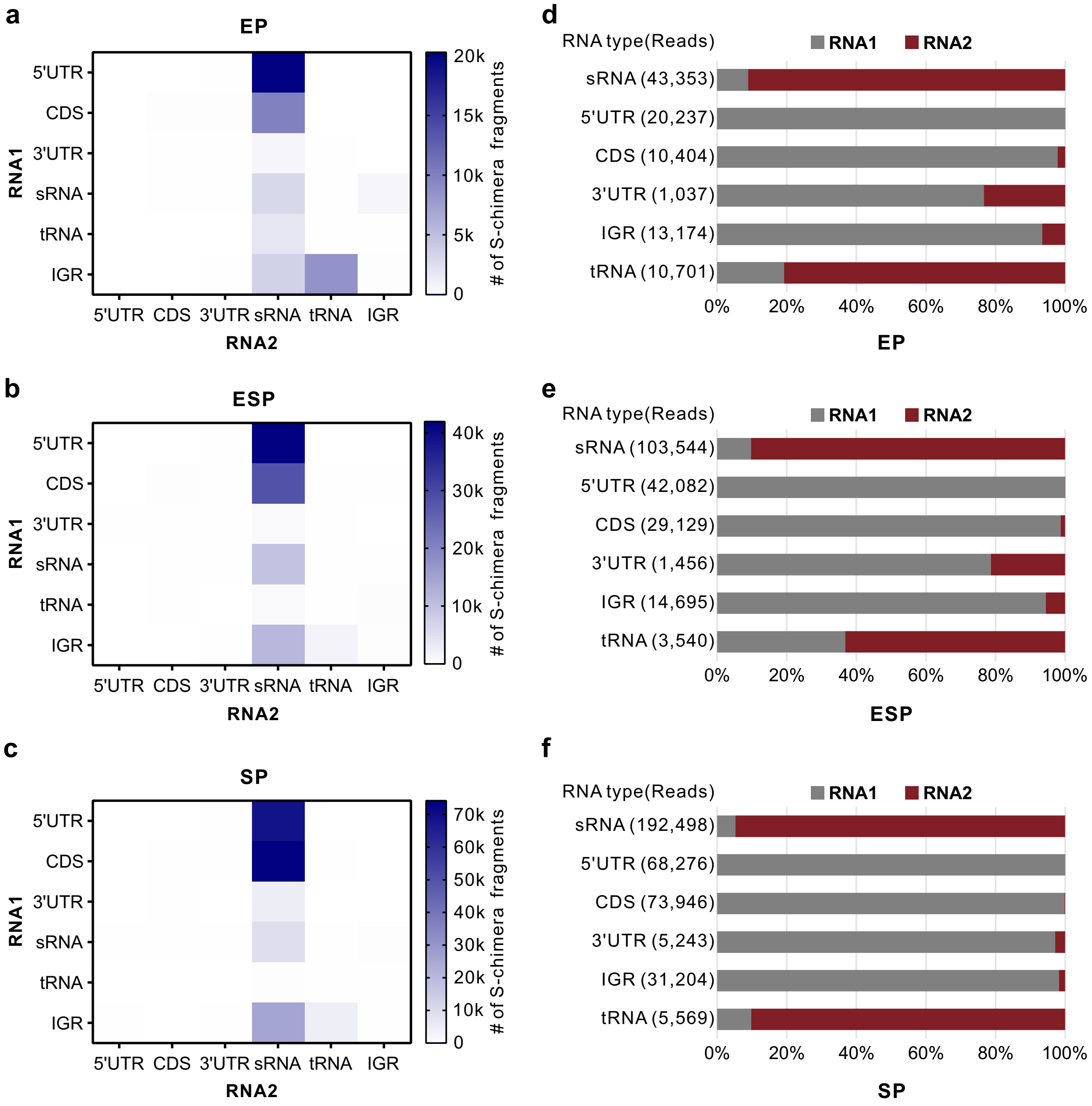

Supplementary Fig. 4 Most S-chimeras are ligation products between mRNA (RNA1) and sRNA (RNA2). a-c Abundance of S-chimera fragments for each combination of transcript type at three growth stages. RNA1, the 5’ terminal RNA in the S-chimera. RNA2, the 3’ terminal RNA in the S-chimera. **d-f** Distribution of RNA1 and RNA2 in S-chimeric fragments for each transcript type at three growth stages.

Supplementary Figure 5

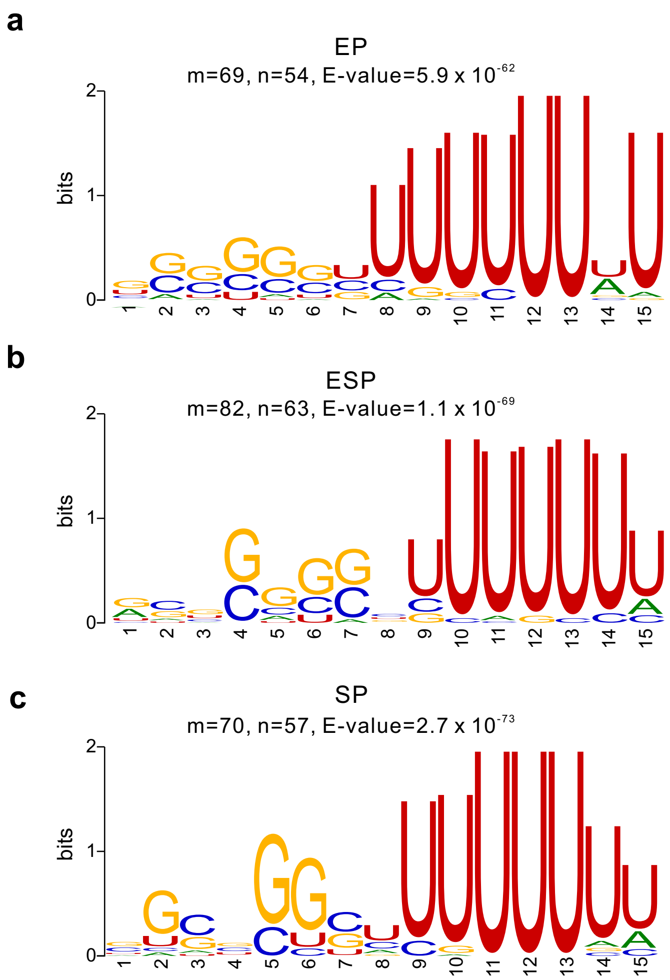

Supplementary Fig. 5 Sequence motifs found in RNA2 of S-chimeras. a-c Sequence motifs are detected in RNA2 in the S-chimeras at three growth stages. m, the number of RNA2 sequences. n, the number of RNA2 sequences that contain the motif. E-value, the statistical significance of the motif.

Supplementary Figure 6

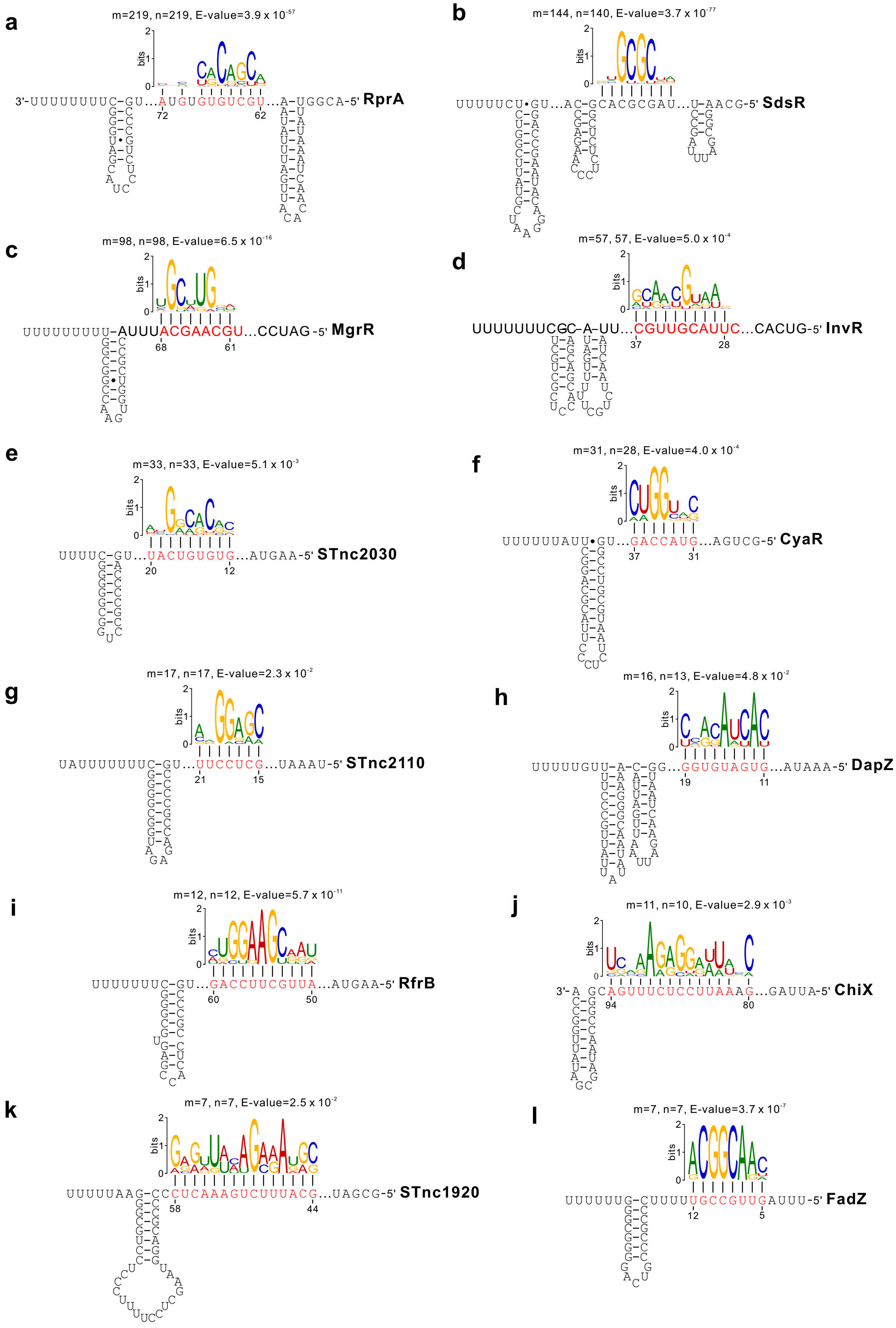

Supplementary Fig. 6 Sequence motifs shared by sRNA targets in S-chimeras. a-l Sequence motifs found in the target genes are complementary to the cognate sRNAs. m, the number of target sequences. n, the number of target sequences that contain the motif. E-value, the statistical significance of the motif.

Supplementary Figure 7

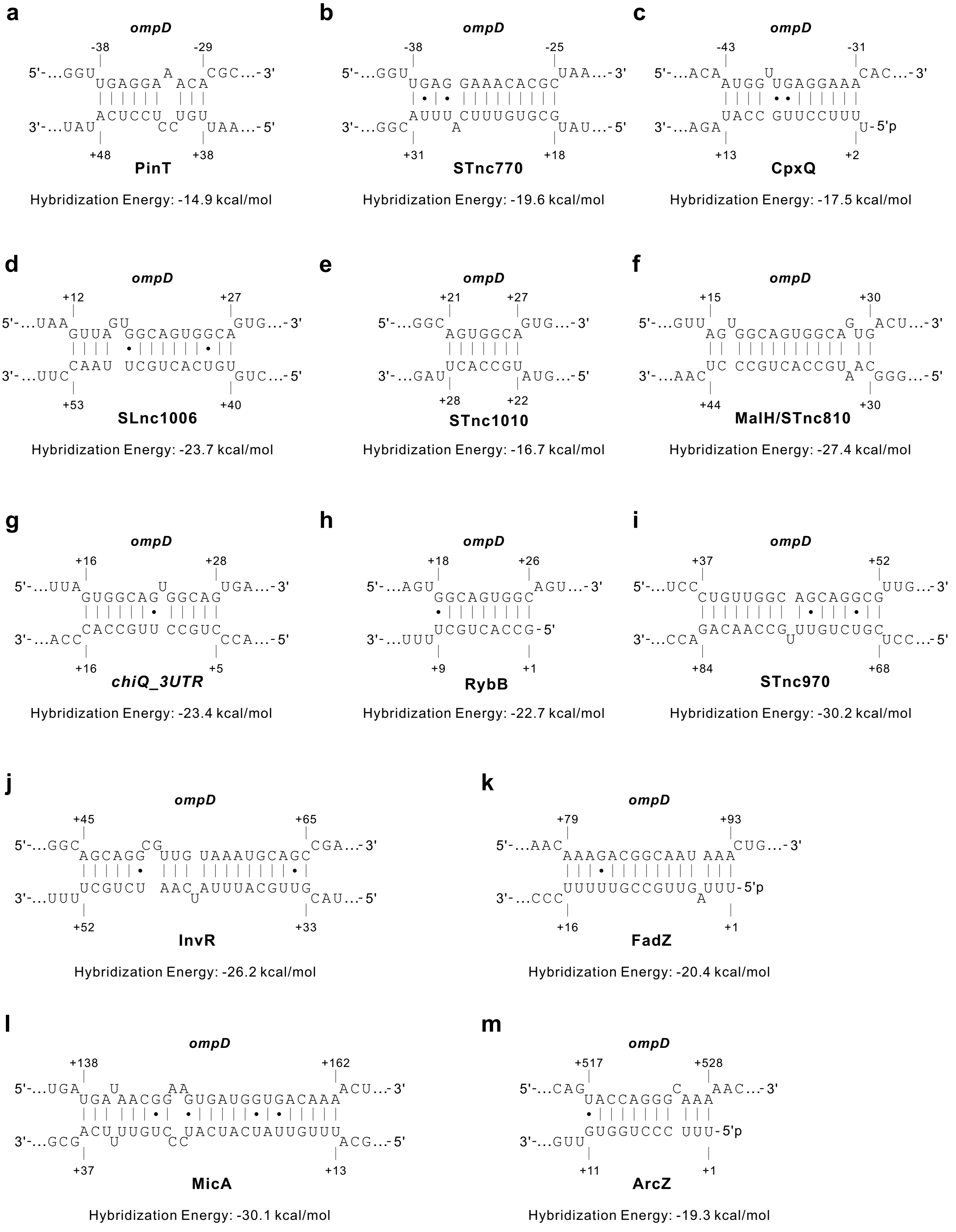

Supplementary Fig. 7 Predicted base-parings between the *ompD* mRNA and sRNAs. a-m Base-paring sites are predicted by RNAhybrid using the sRNA sequence and the *ompD* sequence detected in S-chimeras.

Supplementary Figure 8

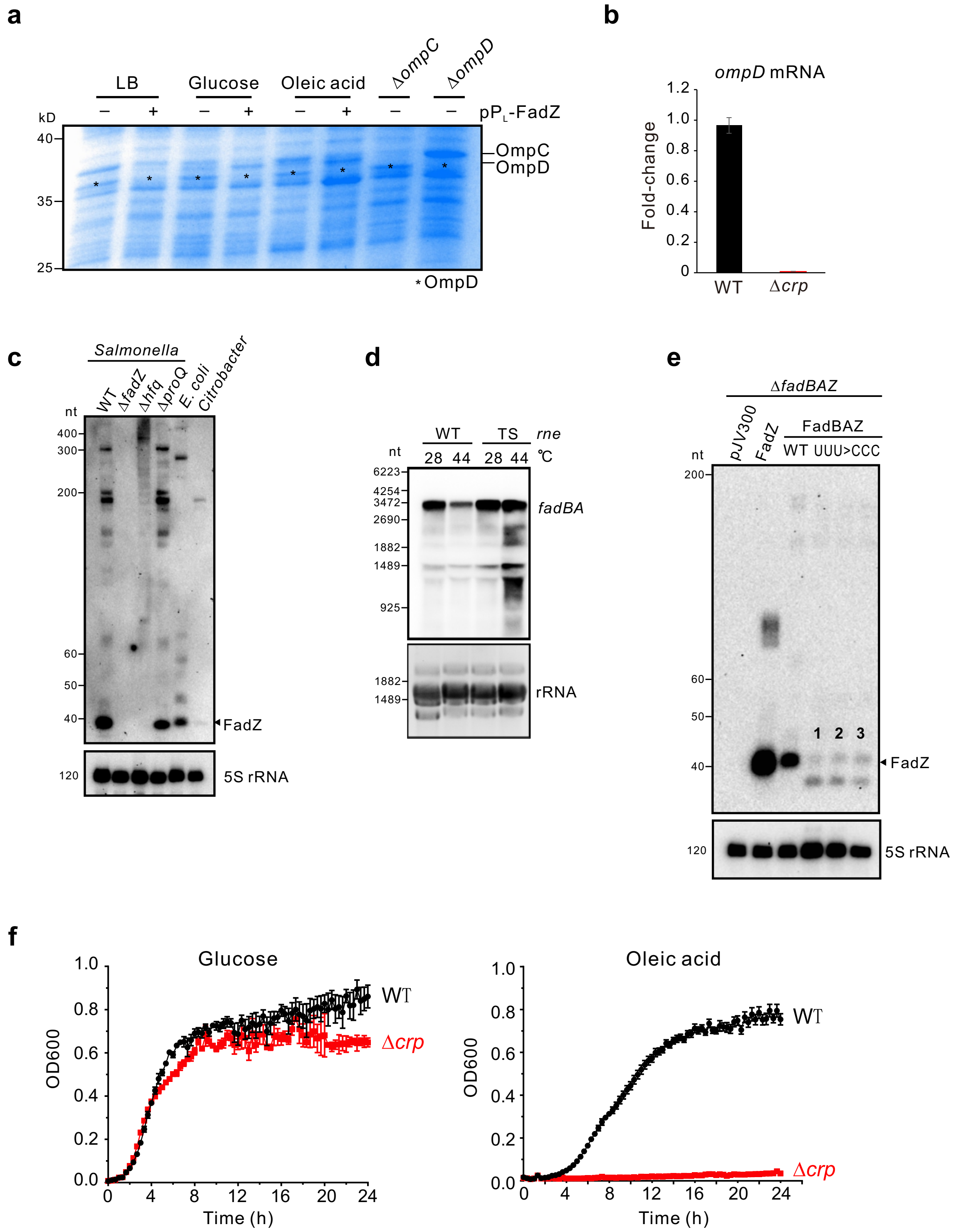

Supplementary Fig. 8 FadZ is a 3'UTR-processed sRNA in fatty acid metabolism pathway. a SDS-PAGE analysis of total proteins from bacteria grown in different media. **b** The *ompD* mRNA level was quantified by qRT-PCR, in WT and Δ*crp* mutant. **c** *Salmonella* mutants were grown overnight in LB, and total RNAs were analyzed for FadZ expression by northern blotting. **d** Total RNA samples were separated on a 1% agarose gel under denatured conditions. Northern blotting was performed to detect the *fadBA* mRNA in the temperature-sensitive *rne* strains. **e** A 'UUU' to 'CCC' mutation was introduced at the RNase E cleavage site in the *fadBAZ* mRNA. The FadZ sRNA was expressed from the constitutive $P_{LlacO\text{-}1}$ promoter. The FadBAZ variants was driven by their native promoter, whereas pJV300 serves as a negative control. Total RNAs were analyzed using northern blotting. **f** The growth curves of *Salmonella* WT and Δ*crp* mutant in M9CA media containing either 0.1% glucose or 0.1% oleic acid as sole carbon sources. Data points represent average OD_{600} value. Error bars indicate standard deviations form *n* = 3 biological replicates. Source data are provided as a Source Data file.

Supplementary Data

Supplementary Data 1. Bacterial strains used in this study.

Supplementary Data 2. Plasmids used in this study.

Supplementary Data 3. Oligonucleotides used in this study.

Supplementary Data 4. Number of Illumina reads from sequencing libraries.

Supplementary Data 5. List of all S-chimeras at three growth stages.

Supplementary Data 6. List of known processed sRNAs used in Figure 3G.

Supplementary Data 7. Genes with 3’UTRs located in RNA2 in S-chimeras.
