# Supplementary material for: In vivo RNA interactome profiling reveals 3’UTR-processed small RNA targeting a central regulatory hub
Source: Nat Commun. 2023 Dec 7;14:8106. doi: 10.1038/s41467-023-43632-1 (PMC10703908; doi:10.1038/s41467-023-43632-1)
Supplement: Supplementary file 3 — Reporting Summary [file 41467_2023_43632_MOESM3_ESM.pdf]

nature portfolio

Corresponding author(s): Yanjie Chao
Last updated by author(s): Nov 10, 2023

Reporting Summary

Nature Portfolio wishes to improve the reproducibility of the work that we publish. This form provides structure for consistency and transparency in reporting. For further information on Nature Portfolio policies, see our Editorial Policies and the Editorial Policy Checklist.

Statistics

For all statistical analyses, confirm that the following items are present in the figure legend, table legend, main text, or Methods section.

n/a	Confirmed	
☐	☒	The exact sample size (*n*) for each experimental group/condition, given as a discrete number and unit of measurement
☐	☒	A statement on whether measurements were taken from distinct samples or whether the same sample was measured repeatedly
☐	☒	The statistical test(s) used AND whether they are one- or two-sided<br>*Only common tests should be described solely by name; describe more complex techniques in the Methods section.*
☒	☐	A description of all covariates tested
☒	☐	A description of any assumptions or corrections, such as tests of normality and adjustment for multiple comparisons
☐	☒	A full description of the statistical parameters including central tendency (e.g. means) or other basic estimates (e.g. regression coefficient) AND variation (e.g. standard deviation) or associated estimates of uncertainty (e.g. confidence intervals)
☐	☒	For null hypothesis testing, the test statistic (e.g. *F*, *t*, *r*) with confidence intervals, effect sizes, degrees of freedom and *P* value noted<br>*Give P values as exact values whenever suitable.*
☒	☐	For Bayesian analysis, information on the choice of priors and Markov chain Monte Carlo settings
☒	☐	For hierarchical and complex designs, identification of the appropriate level for tests and full reporting of outcomes
☒	☐	Estimates of effect sizes (e.g. Cohen's *d*, Pearson's *r*), indicating how they were calculated

Our web collection on statistics for biologists contains articles on many of the points above.

Software and code

Policy information about availability of computer code

Data collection	NovaSeq 6000 (Illumina)
Data analysis	No custom algorithms or softwares was used. The analysis pipelines in this study employ tools that have been previously described in published literature and are described below. The RNA-seq data was analyzed using open-source softwares including Cutadapt (Version 2.10), FASTXToolkit (Version 0.0.13), BWA (Version 0.7.17-r1198-dirty), and MEME (Version 5.5.4). The statistics was analyzed using GraphPad (version 9).

For manuscripts utilizing custom algorithms or software that are central to the research but not yet described in published literature, software must be made available to editors and reviewers. We strongly encourage code deposition in a community repository (e.g. GitHub). See the Nature Portfolio guidelines for submitting code & software for further information.

Data

Policy information about availability of data

All manuscripts must include a data availability statement. This statement should provide the following information, where applicable:

- Accession codes, unique identifiers, or web links for publicly available datasets
- A description of any restrictions on data availability
- For clinical datasets or third party data, please ensure that the statement adheres to our policy

The reference genome used for mapping is Salmonella strain SL1344 (NC_016810.1). The sequencing data have been deposited in the GEO database under accession number: GSE234792. Raw data, materials and regents are available upon request.

Research involving human participants, their data, or biological material

Policy information about studies with human participants or human data. See also policy information about sex, gender (identity/presentation), and sexual orientation and race, ethnicity and racism.

Reporting on sex and gender	N/A
Reporting on race, ethnicity, or other socially relevant groupings	N/A
Population characteristics	N/A
Recruitment	N/A
Ethics oversight	N/A

Note that full information on the approval of the study protocol must also be provided in the manuscript.

Field-specific reporting

Please select the one below that is the best fit for your research. If you are not sure, read the appropriate sections before making your selection.

☒ Life sciences ☐ Behavioural & social sciences ☐ Ecological, evolutionary & environmental sciences

For a reference copy of the document with all sections, see nature.com/documents/nr-reporting-summary-flat.pdf

Life sciences study design

All studies must disclose on these points even when the disclosure is negative.

Sample size	No sample-size calculation was performed. Sample size was chosen based on previous experience and standards in the field. At least three colonies were picked as biological replicates for each growth-related assay.
Data exclusions	No data were excluded from the analyses.
Replication	Each experiment was repeated at least three times, and all attempts at replication were successful.
Randomization	Bacterial colonies with the same genetic background were randomly allocated to experimental groups.
Blinding	Blinding was not performed and not relevant in this study. Not applicable as this study did not involve patients or clinical trials.

Reporting for specific materials, systems and methods

We require information from authors about some types of materials, experimental systems and methods used in many studies. Here, indicate whether each material, system or method listed is relevant to your study. If you are not sure if a list item applies to your research, read the appropriate section before selecting a response.

Materials & experimental systems

n/a	Involved in the study
☐	☒ Antibodies
☒	☐ Eukaryotic cell lines
☒	☐ Palaeontology and archaeology
☒	☐ Animals and other organisms
☒	☐ Clinical data
☒	☐ Dual use research of concern
☒	☐ Plants

Methods

n/a	Involved in the study
☒	☐ ChIP-seq
☒	☐ Flow cytometry
☒	☐ MRI-based neuroimaging

Antibodies

Antibodies used	Polyclonal anti-GroEL antibody produced in rabbit, Sigma, #G6532, 1:10000;<br>Polyclonal anti-6-His antibody produced in rabbit, Sigma, #SAB4301134, 1:1,000;<br>Monoclonal anti-FLAG M2 antibody produced in mouse, Sigma, #F1804, 1:10000;<br>Monoclonal anti-GFP antibody produced in mouse, Roche, #11814460001, 1:10000;<br>secondary HRP-linked goat anti-mouse IgG antibody, BBI, #D110087, 1:10000;<br>secondary HRP-linked goat anti-rabbit IgG antibody, BBI, #D110058, 1:10000.
Validation	We used commercial antibody reagents for immunoprecipitation and Western-blot. Validation data are available on manufacturer's websites and data sheets.
