# Supplementary material for: In vivo RNA interactome profiling reveals 3’UTR-processed small RNA targeting a central regulatory hub
Source: Nat Commun. 2023 Dec 7;14:8106. doi: 10.1038/s41467-023-43632-1 (PMC10703908; doi:10.1038/s41467-023-43632-1)
Supplement: Supplementary file 4 — Supplementary Data 1 [file 41467_2023_43632_MOESM4_ESM.docx]

Supplementary Data 1. Bacterial strains used in this study.

| Trivial name | Strain Name | Relevant markers/genotype | Reference |
| --- | --- | --- | --- |
| Wild-type | YCSH-0094 | *Str*^R^ *hisG rpsL xyl* | (Hoiseth and Stocker, 1981), provided by J. Vogel, IMIB Würzburg |
|  | QGS-559 | *E. coli K-12 MG1655* | Lab stock |
|  | QGS-502 | *Citrobacter freundii 3056* | Lab stock |
| Δ*proQ* | QGS-004 | *SL1344* Δ*proQ* | Lab stock |
| Δ*hfq* | QGS-002 | *SL1344* Δ*hfq* | Lab stock |
| *rne-Control* | QGS-009 | *SL1344 (rluC-rne) IG::cat.  IG, intergenic region.* | (Figueroa-Bossi et al. Genes Dev. 2009,23:2004-15) |
| *rne-TS* | QGS-010 | *SL1344 (rluC-rne) IG::cat/ rne-3071 (ts).  IG, intergenic region.* | (Figueroa-Bossi et al. Genes Dev. 2009,23:2004-15) |
| *hfq*-3×FLAG | YCSH-0375 | *SL1344 hfq*-3×FLAG::Kan^R^ | This study |
| Δ*fadZ* | QGS-704 | *SL1344* Δ*fadZ* | This study |
| Δ*fadR* | QGS-695 | *SL1344* Δ*fadR* | This study |
| Δ*fadBAZ* | QGS-694 | *SL1344* Δ*fadBAZ* | This study |
| Δ*ompC* | QGS-1415 | SL1344 Δ*ompC* | This study |
| Δ*ompD* | QGS-1416 | SL1344 Δ*ompD* | This study |
| Δ*crp* | QGS-1276 | *SL1344* Δ*crp* | This study |
|  | YCSH-0047 | *E. coli* TG1, F-, φ80, lacZ ΔM15, Δ (lacZYA-argF) U169, endA1, recA1hsdR17(rk-, mk+), supE44, λ-, thi-1, gyrA96, relA1, phoA | TIANGEN Biotech |
