# Supplementary material for: In vivo RNA interactome profiling reveals 3’UTR-processed small RNA targeting a central regulatory hub
Source: Nat Commun. 2023 Dec 7;14:8106. doi: 10.1038/s41467-023-43632-1 (PMC10703908; doi:10.1038/s41467-023-43632-1)
Supplement: Supplementary file 5 — Supplementary Data 2 [file 41467_2023_43632_MOESM5_ESM.docx]

Supplementary Data 2. Plasmids used in this study.

| Trivial name | Stock name | Relevant fragment | Comment | Origin/  marker | Reference |
| --- | --- | --- | --- | --- | --- |
| vect/pBAD | pYC564 | pYC564 | pBAD control vector, expresses a ~50 nt nonsense transcript derived from rrnB terminator. | pBR322, HyG^R^ | This study |
| pBAD-*t4rnl1* | pYC582 | P_BAD_-*t4rnl1* | T4 RNA ligase inducible expression plasmid. pBAD vector was amplified with YCO-0453/-0454 and followed by XbaI digestion. *T4rnl1* was amplified from pKH13 and ligated into pBAD at the XbaI site. | pBR322, Hyg^R^ | This study |
|  | pKH13 | P_tac_-*t4rnl1* | Plasmid expressing T4 RNA ligase 1. | pBR322, Amp^R^ | Han et al., 2016 |
|  | pKD46 | P_araB_-γ-*β*-*exo* | Temperature-sensitive lambda red recombinase expression plasmid | oriR101, Amp^R^ | Datsenko and Wanner, 2000 |
|  | pSUB11 |  | Template plasmid to creat PCR product with KanR cassette for epitope tagging of chromosomal genes | oriRγ, Amp^R^ | Sergio Uzzau et al. 2001 |
|  | pKD4 |  | Template plasmid to creat PCR product with KanR cassette for gene knock-out | oriRγ, Amp^R^ | Datsenko and Wanner 2000 |
| Empty Vector | pJV300 | pJV300 | Control vector for sRNA expression, expresses a ~50 nt nonsense transcript derived from *rrnB* terminator. | ColE1, Amp^R^ | Sittka et al., 2007 |
| pZE12-luc | pZE12-luc | pZE12-luc | Parental vector for sRNA overexpression, pZE12 backbone was amplified with YCO-0557/-0558 and followed by XbaI digestion. | ColE1, Amp^R^ | Sittka et al., 2007 |
| pZE12-CpxQ | pYC635 | P_LlacO_-*cpxQ* | CpxQ overexpression plasmid. The *cpxQ* locus was amplified with YCO-1029/-0526 and digested by XbaI, and subsequently ligated into pZE12 backbone. | ColE1, Amp^R^ | This study |
| pZE12-ArcZ | pYC636 | P_LlacO_-*arcZ* | ArcZ overexpression plasmid. The *arcZ* locus was amplified with YCO-1211/-1212 and digested by XbaI, and subsequently ligated into pZE12 backbone. | ColE1, Amp^R^ | This study |
| pZE12-PinT | pYC637 | P_LlacO_-*pinT* | PinT overexpression plasmid. The *pinT* locus was amplified with YCO-1290/-1291 and digested by XbaI, and subsequently ligated into pZE12 backbone. | ColE1, Amp^R^ | This study |
| pZE12-MicA | pYC638 | P_LlacO_-*micA* | MicA overexpression plasmid. The *micA* locus was amplified with YCO-1679/-1680 and digested by XbaI, and subsequently ligated into pZE12 backbone. | ColE1, Amp^R^ | This study |
| pZE12-STnc970 | pYC639 | P_LlacO_-*STnc970* | STnc970 overexpression plasmid. The *STnc970* locus was amplified with YCO-1772/-1773 and digested by XbaI, and subsequently ligated into pZE12 backbone. | ColE1, Amp^R^ | This study |
| pZE12-STnc3530 | pYC640 | P_LlacO_-*STnc3530* | STnc3530 overexpression plasmid. The *STnc3530* locus was amplified with YCO-1774/-1775 and digested by XbaI, and subsequently ligated into pZE12 backbone. | ColE1, Amp^R^ | This study |
| pZE12-STnc1010 | pYC641 | P_LlacO_-*STnc1010* | STnc1010 overexpression plasmid. The *STnc1010* locus was amplified with YCO-1776/-1777 and digested by XbaI, and subsequently ligated into pZE12 backbone. | ColE1, Amp^R^ | This study |
| pZE12-STnc770 | pYC642 | P_LlacO_-*STnc770* | STnc770 overexpression plasmid. The *STnc770* locus was amplified with YCO-1778/-1779 and digested by XbaI, and subsequently ligated into pZE12 backbone. | ColE1, Amp^R^ | This study |
| pZE12-MalH | pYC643 | P_LlacO_-*STnc810* | MalH overexpression plasmid. The *malH* locus was amplified with YCO-1827/-1781 and digested by XbaI, and subsequently ligated into pZE12 backbone. | ColE1, Amp^R^ | This study |
| pZE12-ChiQ_3UTR | pYC644 | P_LlacO_-*SL0670_3UTR* | ChiQ_3UTR overexpression plasmid. The *chiQ_3UTR* locus was amplified with YCO-1828/-1829 and digested by XbaI, and subsequently ligated into pZE12 backbone. | ColE1, Amp^R^ | This study |
| pZE12-FadZ | pZE12-FadZ | P_LlacO_-*fadZ* | FadZ overexpression plasmid. The *fadZ* locus was amplified with QGO-1620/-1621 and digested by XbaI, and subsequently ligated into pZE12 backbone. | ColE1, Amp^R^ | This study |
| pZE12-RybB | pZE12-RybB | P_LlacO_-*rybB* | RybB overexpression plasmid. The *rybB* locus was amplified with QGO-2294/-2295 and digested by XbaI, and subsequently ligated into pZE12 backbone. | ColE1, Amp^R^ | This study |
|  | pXG-1 | P_LtetO_-GFP | Control vector containing GFP+. | pSC101*, CmR | Urban and Vogel 2007 |
|  | pXG10-SF | P_LtetO_-sfGFP | Control vector containing wild-type superforlder GFP (sfGFP), and parental vector for construing sfGFP fusions. | pSC101*, CmR | Corcoran et al., 2012 |
| pZE12-FadBAZ | pZE12-FadBAZ | P*_fadBAZ_-fadBAZ* | pZE12-luc was digested with XhoI and XbaI. The P_fadBAZ_-*fadBAZ* locus was amplified with QGO-1600/-1621 and digested by XhoI and XbaI, and subsequently ligated into pZE12 vector. | ColE1, Amp^R^ | This study |
| pZE12-FadBA | pZE12-FadBA | P*_fadBA_-fadBA* | pZE12-luc was digested with XhoI and XbaI. The P_fadBAZ_-*fadBA* locus was amplified with QGO-1600/-1601 and digested by XhoI and XbaI, and subsequently ligated into pZE12 vector. | ColE1, Amp^R^ | This study |
| pZE12-FadZ-M | pZE12-FadZ-M | P_LlacO_-*fadZ* (with mutation) | The *fadZ mutant* locus was amplified with QGO-2457/-2458 and digested by XbaI, and subsequently ligated into pZE12 backbone. | ColE1, Amp^R^ | This study |
| pXG10-ompC-sfgfp | pXG10-ompC-sfgfp | P_LtetO_-ompC-35aa-sfgfp | SL1344 *ompC* translational GFP reporter plasmid. pXG10-sf was digested with NheI and NsiI. OmpC-35aa was cloned into pXG10-sf by PCR with QGO-1568/-2310 via same digestions. | pSC101*, CmR | This study |
| pXG10-ompD-sfgfp | pXG10-ompD-sfgfp | P_LtetO_-ompD-35aa-sfgfp | SL1344 *ompD* translational GFP reporter plasmid. pXG10-sf was digested with NheI and NsiI. OmpD-35aa was cloned into pXG10-sf by PCR with QGO-1584/-2311 via same digestions. | pSC101*, CmR | This study |
| pXG10-ompN-sfgfp | pXG10-ompN-sfgfp | P_LtetO_-ompN-35aa-sfgfp | SL1344 *ompN* translational GFP reporter plasmid. pXG10-sf was digested with NheI and NsiI. OmpN-35aa was cloned into pXG10-sf by PCR with QGO-1574/-2312 via same digestions. | pSC101*, CmR | This study |
| pXG10-ompS-sfgfp | pXG10-ompS-sfgfp | P_LtetO_-ompS-35aa-sfgfp | SL1344 ompS translational GFP reporter plasmid. pXG10-sf was digested with NheI and NsiI. OmpS-35aa was cloned into pXG10-sf by PCR with QGO-2315/-2316 via same digestions. | pSC101*, CmR | This study |
| pXG10-ompC-M-sfgfp | pXG10-ompC-M-sfgfp | P_LtetO_-ompC-35aa-sfgfp (with mutation) | SL1344 *ompC*-mutant translational GFP reporter plasmid. pXG10-sf was digested with NheI and NsiI. OmpC-35aa-mutant was cloned into pXG10-sf by PCR with QGO-1568/-2851 via same digestions. | pSC101*, CmR | This study |
| pXG10-ompD-M-sfgfp | pXG10-ompD-M-sfgfp | P_LtetO_-ompD-35aa-sfgfp (with mutation) | SL1344 ompD-mutant translational GFP reporter plasmid. pXG10-sf was digested with NheI and NsiI. OmpD-35aa-mutant was cloned into pXG10-sf by PCR with QGO-1584/-2849 via same digestions. | pSC101*, CmR | This study |
| pZE12-FadBAZ (UUU>CCC) | pZE12-FadBAZ (UUU>CCC) | P_LlacO_-*FadBAZ* (with mutation) | pZE12-FadBAZ was amplified with QGO-2837/2838 and treated with TaKaRa MutanBEST Kit following the guide. | ColE1, Amp^R^ | This study |
